# Abnormal dynamic functional connectivity of thalamic subregions in patients with first-episode, drug-naïve major depressive disorder

**DOI:** 10.3389/fpsyt.2023.1152332

**Published:** 2023-05-10

**Authors:** Yanting Zheng, Yujie Wu, Yujie Liu, Danian Li, Xinyu Liang, Yaoping Chen, Hanyue Zhang, Yan Guo, Ruoxi Lu, Jinhui Wang, Shijun Qiu

**Affiliations:** ^1^Department of Radiology, The First Affiliated Hospital of Guangzhou University of Chinese Medicine, Guangzhou, Guangdong, China; ^2^Department of Clinical Psychology, Guangdong Provincial People’s Hospital, Guangdong Academy of Medical Sciences, Guangzhou, Guangdong, China; ^3^Department of Radiology, Guangzhou First People’s Hospital, Guangzhou, Guangdong, China; ^4^Cerebropathy Center, The First Affiliated Hospital of Guangzhou University of Chinese Medicine, Guangzhou, Guangdong, China; ^5^The First School of Clinical Medicine, Guangzhou University of Chinese Medicine, Guangzhou, Guangdong, China; ^6^The Third Affiliated Hospital of Sun Yat-sen University, Guangzhou, Guangdong, China; ^7^Institute for Brain Research and Rehabilitation, South China Normal University, Guangzhou, China; ^8^Key Laboratory of Brain, Cognition and Education Sciences, Ministry of Education, Guangzhou, China; ^9^Center for Studies of Psychological Application, South China Normal University, Guangzhou, China; ^10^Guangdong Key Laboratory of Mental Health and Cognitive Science, South China Normal University, Guangzhou, China

**Keywords:** major depressive disorder (MDD), dynamic functional connectivity, thalamic subregion, childhood trauma questionnaires, magnetic resonance imaging (MRI)

## Abstract

**Background:**

Recent studies have shown that major depressive disorder (MDD) is associated with altered intrinsic functional connectivity (FC) of the thalamus; however, investigations of these alterations at a finer time scale and the level of thalamic subregions are still lacking.

**Methods:**

We collected resting-state functional MRI data from 100 treatment-naïve, first-episode MDD patients and 99 age-, gender- and education-matched healthy controls (HCs). Seed-based whole-brain sliding window-based dFC analyses were performed for 16 thalamic subregions. Between-group differences in the mean and variance of dFC were determined using threshold-free cluster enhancement algorithm. For significant alterations, there relationships with clinical and neuropsychological variables were further examined via bivariate and multivariate correlation analyses.

**Results:**

Of all thalamic subregions, only the left sensory thalamus (Stha) showed altered variance of dFC in the patients characterized by increases with the left inferior parietal lobule, left superior frontal gyrus, left inferior temporal gyrus, and left precuneus, and decreases with multiple frontal, temporal, parietal, and subcortical regions. These alterations accounted for, to a great extent, clinical, and neuropsychological characteristics of the patients as revealed by the multivariate correlation analysis. In addition, the bivariate correlation analysis revealed a positive correlation between the variance of dFC between the left Stha and right inferior temporal gurus/fusiform and childhood trauma questionnaires scores (*r* = 0.562, *P* < 0.001).

**Conclusion:**

These findings suggest that the left Stha is the most vulnerable thalamic subregion to MDD, whose dFC alterations may serve as potential biomarkers for the diagnosis of the disease.

## 1. Introduction

Major depressive disorder (MDD) is a leading cause of disability and is characterized by persistent negative mood. According to the World Health Organization (WHO), over 300 million people suffer from this disease globally, which leads a huge burden on patients, the healthcare system and society worldwide (1). However, the underlying MDD remain unclear. With the development of technology, multimodal magnetic resonance imaging (MRI) is regarded as an important way for exploring the neural mechanism of MDD (2, 3), which may help a more accurate diagnosis and more effective treatment for MDD.

Among different MRI modalities, resting-state functional MRI (R-fMRI) is a promising technique for measuring spontaneous neural activity as blood oxygen level-dependent signals (4). With this technique, numerous studies have demonstrated that MDD is associated with aberrant functional connectivity (FC) of multiple brain regions, such as the thalamus. The thalamus is a walnut-sized, bilateral structure in the deep brain. It comprises approximately 60 nuclei, each of which has distinct inputs and outputs to cortical, subcortical, and cerebellar regions. Based on the inputs, the thalamic nuclei can be categorized into two groups: first-order and higher-order nuclei (5). The first-order nuclei relay driver inputs from subcortical structures and relay the primary sensory information to the cortex, while the higher-order nuclei receive driver inputs from layer five of upstream cortical regions and relay the information to other cortical sites, forming the cortico-thalamo-cortical loops (6). In this manner, the thalamus is an important relay station of signal transmission in the brain, whose dysconnectivity may contribute to symptoms and disturbances in psychiatric patients, including depressed individuals (7). Specifically, altered thalamic FC has been reported in MDD with the somatosensory areas (8, 9), superior frontal gyrus (10), dorsal anterior cingulate (11), and middle frontal gyrus (12). Moreover, some of the alterations were related to severity of symptoms and neuropsychological functioning of patients (8, 9). These findings collectively suggest that the thalamus is one of the core dysconnectivity nodes in MDD.

Despite previous progress, the altered pattern of thalamic FC is still not well established in MDD. Specifically, previous studies on thalamic FC in MDD mainly utilized static methods to estimate interregional temporal synchronization over the entire scan, which ignore the dynamic and time-varying fluctuations of the temporal synchronization (13). Compared with static FC, dynamic FC (dFC) can characterize network architecture at a finer time scale and thus may provide additional, new insights into connectivity dysconnectivity in MDD (14). Moreover, the previous studies typically considered the thalamus as a unified structure to explore FC alterations in MDD. Obviously, this is contrary to the fact that the thalamus is a complex of structurally and functionally heterogenous subregions, which have different connectivity profiles (15, 16). Therefore, treating the thalamus as a whole may overlook specific and precise FC alterations in MDD. To date, it remains largely unknown whether and how thalamic subregions exhibit differential FC alterations in MDD.

In view of these issues, this study aimed to explore dFC of thalamic subregions in MDD. By performing dFC analysis at the level of thalamic subregion, this study may help establish more specific and precise biomarkers for the objective diagnosis of MDD. Specifically, we collected R-fMRI data from 100 treatment-naïve first-episode MDD patients and 99 healthy controls (HCs). First, a sliding window approach was used to characterize see-based dFC of 16 thalamic subregions in terms of the Human Brainnetome Atlas (16). Then, the mean and variance of dFC of each thalamic subregion over time were calculated and used for between-group comparisons. Finally, for regions showing significant between-group differences, their relationships were examined with clinical variables and neuropsychological tests.

## 2. Materials and methods

### 2.1. Participants

A total of 100 treatment-naïve first episode depression MDD patients and 99 age-, gender-, and education-matched HCs were enrolled in this study. All participants were recruited from the department of Psychiatry at the First Affiliated Hospital of Guangzhou University of Chinese Medicine, Guangzhou, China. All patients were diagnosed by two experienced attending psychiatrists and met the Diagnostic and Statistical Manual of Mental Disorders, Fifth Edition (DSM-V) criteria using Structured Clinical Interview (SCID) (17). All participants were Han Chinese aged between 18 and 55 years old. We used the 17-item Hamilton Depression Rating Scale (HAMD-17) (18) to evaluate illness severity. All patients scored at least 18 on the HAMD, and were at their first episode of depression without any treatment, including antidepressants, psychotherapy, and so on. None of the patients had a history of neurological illness or other psychiatric disorders (e.g., bipolar disorder or schizophrenia). All HCs scored less than seven on the HAMD. Participants were excluded if they had significant systemic or neurologic illness, alcohol or drug abuse, or any contraindications for MRI. All participants had normal findings on conventional MRI, as evaluated by two experienced attending radiologists. This study was conducted in accordance with the principles of Declaration of Helsinki. All participants provided written informed consent, and the study was approved by the Ethics Committee of the First Affiliated Hospital of Guangzhou University of Chinese Medicine, Guangzhou, China (N0. JY2019069).

### 2.2. Neuropsychological tests and clinical variables

Out of all participants, 67 (34 patients and 33 HCs) completed a battery of neuropsychological tests, including the Stroop Interference Effect (SIE), Symbol Digit Modalities Test (SDMT), and Verbal Fluency Test (VFT). These tests can reflect the working memory, cognitive control, executive function, and attention of each participant (19). In particular, we recorded reaction time (SIE-time) and number correct (SIE-accuracy) for the congruent and incongruent tasks. For the VFT, the number of words produced by each subject in his or her 60s were recorded as following: animal (VFT-animal), words begin with “fa” (VFT-fa), and what can we do in the kitchen (VFT-kitchen). In addition, the subset of participants completed the State-trait Anxiety Inventory (STAI) and Beck Depression Rating Scale (BDI) to assess their anxiety and depression state, as well as the Childhood trauma questionnaire (CTQ) to examine childhood trauma experience with respect to abuse and neglect. For each patient, the HAMD score and illness duration were collected.

### 2.3. Image acquisition

All participants were scanned using a 3.0 Tesla GE Signa HDxt scanner with an 8-channel head coil at the Department of Radiology, The First Affiliated Hospital of Guangzhou University of Chinese Medicine. All participants were instructed to keep their eyes closed and remain awake without thinking about anything in particular. Of note, the imaging parameters of the R-fMRI data differed slightly between the participants that completed (termed Dataset 1) and did not completed (termed Dataset 2) the neuropsychological tests. Specifically, the R-fMRI images were obtained using a single-shot gradient-echo echo-planar imaging sequence with the following parameters for the Dataset 1/2: repetition time (TR) = 2,000/2,000 ms, echo time (TE) = 30/30 ms, flip angle (FA) = 90°/90°, 36/33 slices, matrix = 64 × 64/64 × 64, field of view (FOV) = 220 mm × 220 mm/240 mm × 240 mm, slice thickness = 3/4 mm, voxel size = 3.44 mm × 3.44 mm × 3 mm/3.75 mm × 3.75 mm × 4 mm^3^, and scanning time = 370/500 s (i.e., 185/250 volumes). For registration purpose, individual high-resolution T1-weighted images were also acquired using a 3-dimension Bravo sequence with the following parameters for the Dataset 1/2: TR = 6.9/10.4 ms, TE = 1.5/4.3 ms, FA = 12°/15°, 188/156 slices, matrix = 256 × 256/256 × 256, FOV = 256 mm × 256 mm/256 mm × 256 mm, slice thickness = 1.0/1.0 mm, and voxel size = 1 mm × 1 mm × 1 mm/1 mm × 1 × 1 mm.

### 2.4. Data preprocessing

The same preprocessing steps were performed for the R-fMRI images of both the Dataset 1 and Dataset 2 using the GRETNA toolbox (20) based on the SPM12 package.^1^ First, the first five volumes were deleted for each participant. Then, slice timing and head motion correction were done for individual functional images. No participant was excluded because of excessive motion in terms of the criteria of >2 mm in translation, >2 in rotation or >0.5 mm in mean frame-wise displacement. There were no significant between-group differences in the maximum (HCs: 1.663 ± 0.339 mm; MDD: 1.924 ± 0.338 mm; *P* = 0.722, two-sample *t*-test) or mean frame-wise displacement (HCs: 0.094 ± 0.044 mm; MDD: 0.095 ± 0.054 mm; *P* = 0.948, two-sample *t*-test) for head motion. After slice timing and head motion correction, the functional images were further spatially normalized to the standard Montreal Neurological Institute space via applying deformation fields derived from tissue segment of structural images, followed by spatial smoothing (Gaussian kernel with 6-mm FWHM) and temporal band-pass filtering (0.01–0.08 Hz). Finally, several nuisance signals were regressed out from each voxel’s time series, including 24-parameter head motion profiles (21), white matter signals, cerebrospinal fluid signals, and global signals. Of note, all nuisance signals were also band-pass filtered (0.01–0.08 Hz) to avoid reintroducing frequency components of non-interest (22).

### 2.5. Definition of thalamic subregions and calculation of dynamic functional connectivity

**FIGURE 1:**
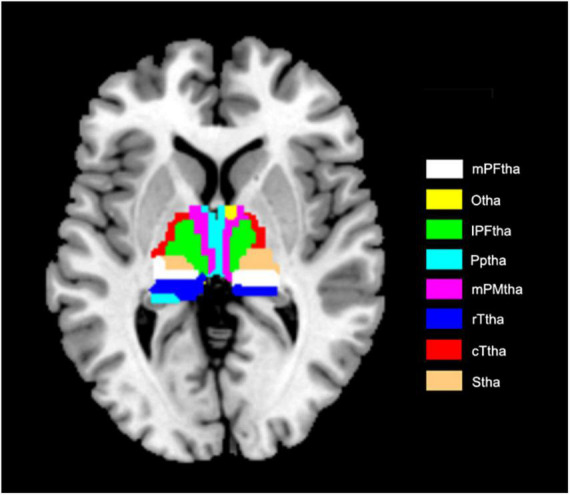
Axial presentation of thalamic subregions. Axial presentation of thalamic subregions. mPFtha, medial Pre-frontal thalamus; Otha, occipital thalamus; IPFtha, lateral pre-frontal thalamus; Pptha, posterior parietal thalamus; mPMtha, pre-motor thalamus; rTtha, rostral temporal thalamus; cTtha, caudal temporal thalamus; Stha, sensory thalamus.

Thalamic subregions were determined using the Human Brainnetome Atlas (16) and included the medial prefrontal thalamus (mPFtha), occipital thalamus (Otha), lateral pre-frontal thalamus (IPFtha), posterior parietal thalamus (PPtha), pre-motor thalamus (mPMtha), rostral temporal thalamus (rTtha), occipital thalamus (cTtha), and sensory thalamus (Stha) in each hemisphere (Figure 1). For each thalamic subregion, we applied a sliding window approach to calculate its dFC for each participant. Following the rule of thumb proposed by Leonardi and Van De Ville (23) and consistent with our previous study (14), the sliding window length was set as 50 TR (100 s) with a step of 1 TR (2 s) in this study. This resulted in 131 time windows for the Dataset 1 and 196 time windows for the Dataset 2. To facilitate cross-dataset consistency, the last 65 time windows were deleted for each participant in the Dataset 2. For each of the remaining time windows, the mean time series was extracted for each thalamic subregion, and correlated with the time series of each voxel in the brain. This generated a total of 16 (seeds) × 131 (time windows) whole-brain FC maps for each participant. These analyses were implemented with the GRETNA toolbox (20).

To extract summary statistics to characterize the dFC maps, we calculated the mean and variance of the dFC maps across all time windows for each thalamic subregion of each participant, which reflect the degree of strength and fluctuation of thalamic FC during the entire scan, respectively. These two metrics not only have genetic bases (24) but also are demonstrated effective to reveal dFC alterations in various brain diseases (e.g., (25–28)).

### 2.6. Removal of effects of different imaging parameters on thalamic dFC

Before calculating the mean and variance of dFC of thalamic subregions, we utilized the ComBat harmonization approach (29) to control for potential effects of different imaging parameters on thalamic FC derived from each time window. Specifically, for a given FC or edge *v* linking a thalamic subregion and a voxel, the Combat model can be written as:


(1) $$y_{i\,j\,v}=\alpha_{v}+X_{i\,j}\,\beta_{v}+\gamma_{i\,v}+\delta_{i\,v}\,\varepsilon_{i\,j\,v}$$

where *y*_*ijv*_ represents the FC strength of the edge *v* for subject *j* in dataset *i*, α_*v*_ is the average FC strength for the edge *v*, *X* is a design matrix for the covariates of interest (e.g., age, sex, and group), β_*v*_ is a vector of regression coefficients corresponding to covariates in *X*, and ε_*ijv*_ is the residual term that is assumed to follow a normal distribution with zero mean. The terms γ_*iv*_ and δ_*iv*_ represent the additive and multiplicative dataset effects of dataset *i* on the edge *v*, respectively. The final ComBat-harmonized FC strength for the edge *v* is calculated as:


(2) $$y_{i\,j\,v}^{C\,o\,m\,B\,a\,t}=\frac{y_{i\,j\,v}-{\hat{\alpha }}_{v}-X_{i\,j}{\hat{\beta }}_{v}-\gamma {*}_{i\,v}}{\delta {*}_{i\,v}}+{\hat{\alpha }}_{v}+X_{i\,j}\,{\hat{\beta }}_{v}$$

where $\gamma_{i\,v}^{*}$and $\delta_{i\,v}^{*}$ are the empirical Bayes estimates of γ_*iv*_ and δ_*iv*_, respectively. These analyses were conducted using public MATLAB codes in GitHub.^2^

### 2.7. Statistical analysis

Demographic and clinical variables were compared between the two groups using a two-sample *t*-test or a chi-square test. Statistical significance was set at *P*-value <0.05 (two tailed). Between-group differences in the mean and variance of thalamic dFC were inferred for each subregion using voxel wise two-sample *t*-test with gender, age, education and head motion as covariates. The resulting *t*-statistic maps were further enhanced via a threshold-free cluster enhancement method (30). Significant levels of enhanced *t*-statistic to maps were estimated through a non-parametric permutation testing procedure (5,000 times) and corrected for multiple comparisons by comparing each voxel’s *t*-statistic to the distribution of the maximum *t*-statistic of all voxels under the null hypothesis. *P* < 0.05 was considered as significant differences.

For regions showing significant alterations in the MDD patients, we further examined their relationships with clinical variables (HAMD scores and disease duration) among all patients via partial correlation analysis. The partial correlation analysis was also used to examine the relationship of altered thalamic dFC with the STAI, BDI, SIE, VFT, SDMT, and CTQ among the patients in the Dataset 1. A false discovery rate (FDR) procedure was used to correct for multiple comparisons at the level of *q* < 0.05. Given the small sample size for the Dataset 1, we additionally utilized canonical correlation analysis (CCA), a multivariate approach for investigating the linear relationship between two sets of variables, to explore the relationship between altered thalamic dFC and other variables (STAI, BDI, SIE, VFT, SDMT, and CTQ) for the patients in the Dataset 1. Effects of gender, age, education, and head motion were controlled in all correlation analysis.

## 3. Results

### 3.1. Demographic and clinical data

The demographic and clinical data of all participants are summarized in Table 1. No significant differences were observed between the two groups in age, gender or education (all *P* > 0.05). Patients with MDD showed higher STAI, BDI, CTQ, and SDMT scores than did the HCs (*P* < 0.05, FDR corrected), as shown in Table 2.

**TABLE 1:** Demographic and clinical characteristics of all participants.

Characteristics	MDD (*n* = 100)	HCs (*n* = 99)	Statistics (*z/χ*^2^)	*P-*value
Age (years)	27 (13)	25 (10.5)	0.282	0.778
Sex (male/female)	34/66	41/58	1.04	0.307
Education (years)	12 (6)	13 (3.5)	−1.053	0.293
HAMD-17 score	22 (4)	–	–	–
Duration (months)	5.5 (11)	–	–	–

Data are represented as median (interquartile range) except for sex. MDD, major depressive disorder; HCs, healthy controls; HAMD, Hamilton depression rating scale.

**TABLE 2:** Neuropsychological tests of a subset of participants.

Characteristics	MDD (*n* = 34)	HCs (*n* = 33)	Statistics (*z/t)*	*P-*value
STAI_state	49.47 ± 11.59	31.47 ± 8.43	53.83	**<0.001**
STAI_trait	55.47 ± 9.06	33.52 ± 8.35	112.56	**<0.001**
BDI	22.67 ± 9.87	4.94 ± 5.73	83.74	**<0.001**
SIE_time	30 (16)	22.5 (9)	1.848	0.065
SIE_accuracy	−2 (4)	−1 (2)	−1.654	0.098
VFT_animal	18.14 ± 5.77	21.47 ± 4.82	5.95	0.017
VFT_fa	7.64 ± 4.74	9.91 ± 3.98	5.41	0.023
VFT_kitchen	12 (5)	14.5 (5)	−2.301	0.021
SDMT	53.21 ± 12.27	62.03 ± 14.12	11.57	**0.001**
CTQ	42.5 (21)	31 (9)	4.562	**<0.001**

Data are represented as mean ± standard deviation or median (interquartile range) depending on whether they are normally distributed. Bold fonts indicate significant between-group differences after multiple comparison correction. MDD, major depressive disorder; HCs, healthy controls; STAI_state, state trait anxiety inventory: state anxiety; STAI_trait, state trait anxiety inventory: trait anxiety; BDI, beck depression inventory; SIE_time, stroop interference effect: reaction time; SIE_accuracy, stroop interference effect: correct number; VFT_animal, verbal fluency test: words of animals; VFT_fa, verbal fluency test: words that begin with “fa”; VFT_kitchen, verbal fluency test: words describing what we can do in the kitchen; SDMT, symbol digit modalities test; and CTQ, childhood trauma questionnaires.

### 3.2. Altered thalamic dFC in MDD

**FIGURE 2:**
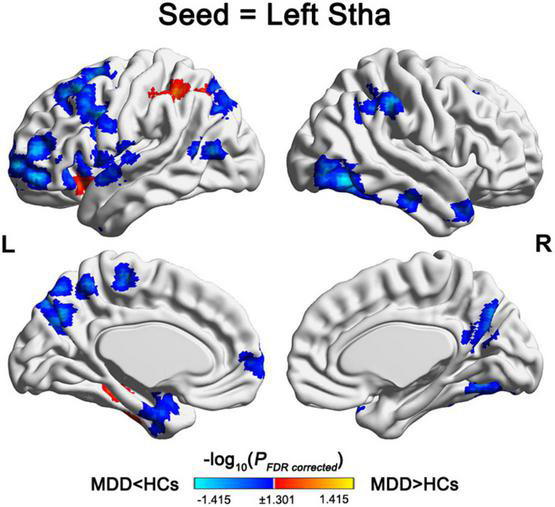
Regions showing alterations in the variance of dFC with the left Stha. For the left Stha, the MDD patients exhibited increased variance of dFC with the left inferior parietal lobule, left superior frontal gyrus, left inferior temporal gyrus, and left precuneus, and decreased variance of dFC with multiple frontal, temporal, parietal, and subcortical regions compared with the HCs. Stha, sensory thalamus; MDD, major depressive disorder; and HCs, health controls.

No significant between-group differences were observed in the mean of thalamic dFC for any subregions. For the variance of the thalamic dFC, significant between-group differences were observed only for the left Stha, characterized by increases with the left inferior parietal lobule, left superior frontal gyrus, left inferior temporal gyrus, left precuneus, and decreases with several frontal (bilateral middle frontal gyrus, bilateral inferior frontal gyrus, left paracentral lobule, and left precentral gyrus), temporal (bilateral superior temporal gyrus, left middle temporal gyrus, and right inferior temporal gyrus), parietal (bilateral precuneus, right inferior parietal lobule, and right supramarginal gyrus), and the subcortical (left insula, left parahippocampal gyrus, and left putamen) regions in the MDD patients compared with the HCs (Table 3 and Figure 2).

**TABLE 3:** Regions showing alterations in the variance of dFC with the left Stha.

Seed	Region	Cluster size (voxels)	Peak coordinates (*X, Y, Z*)
**MDD > HCs**			
Left Stha	Left inferior parietal lobule	78	−42, −36, 36
Left Stha	Left superior frontal gyrus	44	−21, 60, −3
Left Stha	Left inferior temporal gyrus	21	−48, −21, −27
Left Stha	Left precuneus	14	−3, −54, 57
**MDD < HCs**			
Left Stha	Left middle frontal gyrus/precentral gyrus	261	−48, 3, 21
Left Stha	Right inferior temporal gyrus/fusiform	161	57, −51, −24
Left Stha	Right precuneus	71	6, −66, 21
Left Stha	Left middle frontal gyrus	65	−42, 45, −9
Left Stha	Left insula	59	−48, −3, −9
Left Stha	Left parahippocampa gyrus	50	−27, −9, −45
Left Stha	Left precuneus	47	−36, −72, 33
Left Stha	Left inferior frontal gyrus	39	−45, 27, −12
Left Stha	Right supramarginal gyrus	33	54, −39, 39
Left Stha	Left middle temporal gyrus	30	−48, −75, 9
Left Stha	Right inferior parietal lobule	23	39, −42, 39
Left Stha	Left superior temporal gyrus	20	−60, −15, 6
Left Stha	Right middle frontal gyrus	18	27, 15, 42
Left Stha	Left inferior frontal gyrus	14	−36, 12, −12
Left Stha	Left putamen	14	−24, 6, −3
Left Stha	Right inferior temporal gyrus	13	63, −24, −24
Left Stha	Left precuneus	13	−12, −78, 27
Left Stha	Right superior temporal gyrus	10	48, 6, −33
Left Stha	Left paracentral lobule	10	−6, −27, 57

Stha, sensory thalamus; MDD, major depressive disorder; HCs, healthy controls.

### 3.3. Correlation between thalamic dFC alterations and clinical and neuropsychological variables

**FIGURE 3:**
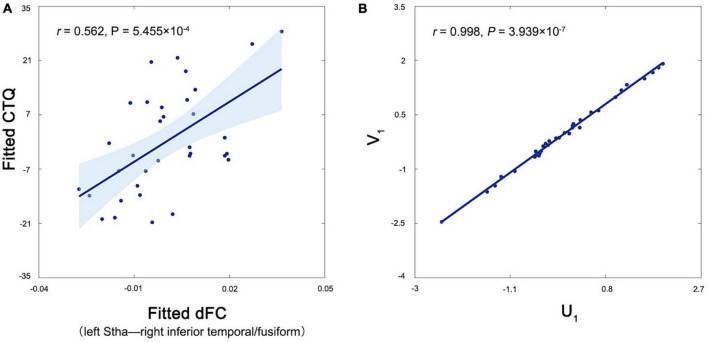
Correlations between the variance of dFC of the left Stha and clinical and neuropsychological variables in the patients. **(A)** The variance of dFC between the left Stha and right inferior temporal gyrus/fusiform was positively correlated with the CTQ score in the patients. **(B)** The CCA revealed a significant positive correlation between the first pair of CCA modes derived separately from all altered dFC of the left Stha (U_1_), and clinical and neuropsychological variables (V_1_) in the patients. Stha, sensory thalamus; dFC, dynamic functional connectivity; and CTQ, childhood trauma questionnaire.

**FIGURE 4:**
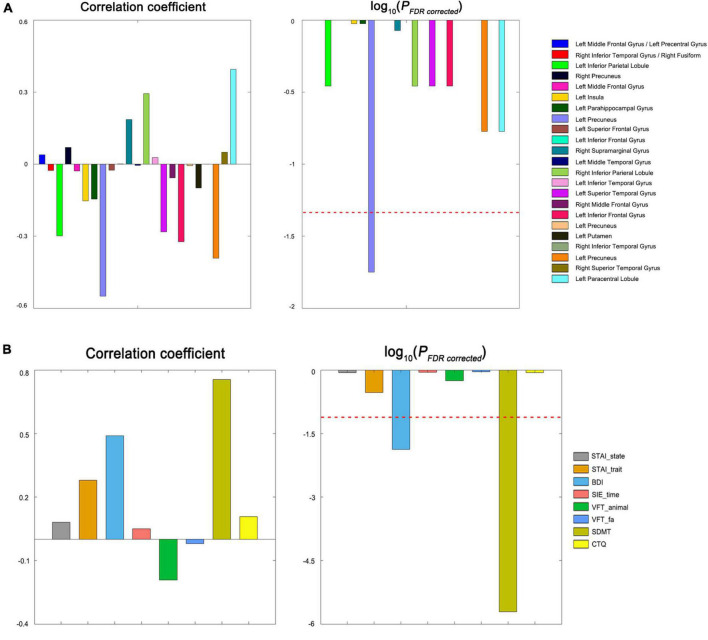
*Post-hoc* correlation results of the CCA. **(A)** The thalamic dFC-related CCA mode (U_1_) was significantly correlated with the variance of dFC between the left Stha and precuneus in the patients. **(B)** The clinical and neuropsychological variables-related CCA mode (V_1_) was significantly correlated with the BDI and SDMT scores patients. STAI, state-trait anxiety inventory; BDI, beck depression rating scale; SIE, stroop interference effect; VFT, verbal fluency test; SDMT, symbol digit modalities test; CTQ, childhood trauma questionnaire; and FDR, false discovery rate.

Among all patients, no significant correlations were observed for the altered variance of thalamic dFC with the HAMD scores or disease duration (*P* > 0.05). For the patients in the Dataset 1, the variance of dFC between the left Stha and right inferior temporal/fusiform was positively correlated with the CTQ scores (*r* = 0.562, *P* < 0.001, FDR corrected) (Figure 3A). The CCA revealed a significant positive correlation between the first pair of CCA modes derived separately from all altered thalamic dFC, and clinical and neuropsychological variables (*r* = 0.998, *P* < 0.001) (Figure 3B). For each CCA mode, we further correlated it with each of its corresponding set of variables. We found that the thalamic dFC-related CCA mode was significantly correlated with the variance of dFC between the left Stha and precuneus, and the other CCA mode was significantly correlated with the BDI and SDMT scores (*P* < 0.05, FDR corrected). These *post-hoc* correlation results (both correlation coefficients and associated *P*-values) are shown in the Figure 4.

## 4. Discussion

To our knowledge, this is the first attempt to study time-varying FC alterations of thalamic subregions in first episode, treatment-naïve depressed patients. We found that the left Stha was the only subregion that showed aberrant dFC with widespread regions in MDD. Moreover, the aberrant dFC of the left Stha was able to account for clinical and neuropsychological characteristics of the patients. These findings suggest that the Stha is a key dysconnectivity component of the thalamus in MDD, which may play a core role in the pathology of the disease.

### 4.1. Subregion-specific alterations in thalamic dFC in MDD

The thalamus is an integrative hub for functional brain networks with numerous connections with various cortical regions (9). Previous studies found that MDD patients showed abnormal FC of the thalamus with multiple brain regions, such as the insula (31), primary somatosensory cortex (9) and superior frontal gyrus (10). However, these studies mainly utilized a static FC approach, which assumes that temporal synchronization between brain areas remains relatively stable. Here using a more advanced dFC approach, we found widespread cortical and subcortical regions that exhibited aberrant fluctuations in the temporal synchronization with the thalamus in the MDD patients. More importantly, all the aberrant fluctuations converged to the Stha subregion, suggesting that the Stha is the most vulnerable subregion to MDD, which may play a key role in the pathophysiology of MDD. The Stha is known to be mainly involved in action and executive functions (16). Accordingly, the observed aberrant fluctuations in the FC of the Stha may be related to action and executive dysfunction in patients with MDD.

### 4.2. Altered variance of dFC of the left Stha in MDD

For the dFC of the left Stha, we found increased variance with the left inferior parietal lobule, left superior frontal gyrus, left inferior temporal gyrus and left precuneus, and decreased variance with multiple frontal, temporal, parietal, and subcortical regions in the MDD patients. Variance of dFC measures temporal variability or instability of a connection. The increased and decreased variance thus imply larger and smaller fluctuations in the strength of the corresponding FC in the patients, respectively. Previous studies found that time-varying, dynamic changes in FC are behaviorally and cognitively relevant (32). For example, evidence from both animal and human studies showed that dFC variability was related to different levels of arousal. More specifically, in both monkeys and rats reduced consciousness resulted in decreased dFC variability (33–35), and in humans higher levels of fatigue were associated with more stable dFC, while higher levels of attention were associated with more variable dFC (36). In particular, the dynamic changes in FC may be important neural mechanisms to support cognitive and behavioral flexibility via rapid transitions between brain states or networks (37). In this context, we speculate that the observed dFC, which switch their states or networks too often or too rarely in the patients, may contribute to emotion dysregulation and cognitive deficits that are frequently observed in MDD, such as increased elaboration of negative information and deficits in attention, executive function, memory, and processing speed (38). This speculation sounds reasonable given previous findings that regional network switching rates predicted interindividual variation in working memory, planning/reasoning and amount of sleep (39), and characteristics of subjects’ transition graphs were correlated with cognition and motor functioning (40). Moreover, the correlation results also supported our speculation. Nevertheless, further studies are needed to examine the speculation by using more sophisticated analytical approaches of dFC in the future, such as state analysis and multilayer network models. Overall, our results suggest that the altered variance of dFC between the Stha and widespread cortical and subcortical regions may constitute a neurophysiological basis for the dysfunction of depressed patients to react flexibly to external or internal cognitive demands.

### 4.3. Clinical and neuropsychological relevance of altered thalamic dFC in MDD

Using the multivariate CCA approach, we found that the altered dFC of the Stha was able to account for, to a great extent, clinical and neuropsychological characteristics of the patients. Further *post-hoc* analyses revealed that the strong association was mainly attributable to the altered dFC of the left Stha with the left precuneus and the BDI and SDMT scores. The BDI scores reflect depressive severity and the SDMT scores reflect information processing and psychomotor speed (41). Therefore, our findings imply that the variance of dFC between the left Stha and precuneus may serve as an indicator to monitor the progression of MDD and neuropsychological function of patients. In addition, our bivariate correlation analysis revealed a positive correlation between the variance of dFC between the left Stha and right inferior temporal gyrus/fusiform with the CTQ scores in the patients. The CTQ measures the history of childhood trauma, which is a life-long burden of risk for the development of various psychiatric disorders. Previous studies have found that childhood trauma significantly affected thalamic volume or FC in obsessive-compulsive disorder (42), panic disorder (43), and post-traumatic stress disorder (44). With regard to MDD, a recent study found that participants with lifetime MDD diagnoses reported higher scores in both the CTQ and childhood trauma metric (shorthand for UKB childhood trauma items) than HCs, which were associated with reduced volumes of the thalamus (45). Another study further showed that childhood maltreatment was associated with increased FC between subcortical structures (particularly the thalamus) and other cortical networks (46). These findings together with our results jointly suggest the core roles of the thalamus in the onset of psychosis due to childhood trauma. This speculation is further supported by findings from a transdiagnostic study, which found that the severity of childhood trauma predicted increased global FC of the thalamus (47). In future, a deeper understanding of the roles of the thalamus in the relationship between childhood trauma and its associated lifelong psychiatric sequelae may benefit from longitudinal studies with large sample size.

## 5. Conclusion

In conclusion, the left Stha is the only thalamic subregion that exhibited aberrant dFC in patients with first-episode, drug-naïve MDD. The dFC alterations may serve as potential biomarkers for the objective diagnosis of MDD although independent validation of the reproducibility is needed before their practical application.

## 6. Limitation

Our study had some limitations. First, the interest in the thalamus was based on previous literature showing the clinical relevance of different thalamic subregions in MDD. This means that we did not investigate other deep gray matter structures that are clearly related to MDD. Second, we only included first-episode, drug-naïve MDD patients in this study. It is unclear whether the observed thalamic dFC alterations will persist in the long-term period of the disease or be normalized by therapy. Third, only a subset of the patients completed the clinical scales and neuropsychological tests, which may cause underestimation of clinical and neuropsychological relevance of thalamic dFC alterations. Thus, our correlation results should be explained with caution and validated by future studies. In addition, a previous study found that reduced thalamic static FC was related to suicidal ideation in patients with MDD (48). It is interesting to explore whether the observed thalamic dFC alterations are also related to suicidal risk of MDD patients in the future. Finally, given that the diagnosis of MDD is highly heterogeneous, it is important to investigate whether the observed thalamic dFC alterations are common to different or specific to certain subtypes of MDD.

## Data availability statement

The raw data supporting the conclusions of this article will be made available by the authors, without undue reservation.

## Ethics statement

The studies involving human participants were reviewed and approved by the First Affiliated Hospital of Guangzhou University of Chinese Medicine. The patients/participants provided their written informed consent to participate in this study.

## Author contributions

YZ, YL, XL, YC, HZ, YG, and RL collected the data. YZ wrote this manuscript. YW analyzed the data. DL administered the neuropsychological tests. JW and SQ design of the study and revising the manuscript. All authors have read and approved the final manuscript.

## Footnotes

1
http://www.fil.ion.ucl.ac.uk/spm/software/spm12

2
https://github.com/Jfortin1/ComBatHarmonization/tree/master/Matlab

## References

[1] World Health Organization. *Depression and other common mental disorders: Global health estimates.* Geneva: World Health Organization (2017).

[2] Dai L Zhou H Xu X Zuo Z. Brain structural and functional changes in patients with major depressive disorder: a literature review. *PeerJ.* (2019) 7:e8170. 10.7717/peerj.8170 31803543 PMC6886485

[3] Zhuo C Li G Lin X Jiang D Xu Y Tian H The rise and fall of MRI studies in major depressive disorder. *Transl Psychiatry.* (2019) 9:335. 10.1038/s41398-019-0680-6 31819044 PMC6901449

[4] Fox MD Raichle ME. Spontaneous fluctuations in brain activity observed with functional magnetic resonance imaging. *Nat Rev Neurosci.* (2007) 8:700–11. 10.1038/nrn2201 17704812

[5] Guillery RW. Anatomical evidence concerning the role of the thalamus in corticocortical communication: a brief review. *J Anat.* (1995) 187(Pt 3):583–92. 8586557 PMC1167461

[6] Shepherd GMG Yamawaki N. Untangling the cortico-thalamo-cortical loop: cellular pieces of a knotty circuit puzzle. *Nat Rev Neurosci.* (2021) 22:389–406. 10.1038/s41583-021-00459-3 33958775 PMC9006917

[7] Hwang WJ Kwak YB Cho KIK Lee TY Oh H Ha M Thalamic connectivity system across psychiatric disorders: current status and clinical implications. *Biol Psychiatry Glob Open Sci.* (2022) 2:332–40. 10.1016/j.bpsgos.2021.09.008 36324665 PMC9616255

[8] Brown EC Clark DL Hassel S Macqueen G Ramasubbu R. Thalamocortical connectivity in major depressive disorder. *J Affect Disord.* (2017) 217:125–31. 10.1016/j.jad.2017.04.004 28407555

[9] Kang L Zhang A Sun N Liu P Yang C Li G Functional connectivity between the thalamus and the primary somatosensory cortex in major depressive disorder: a resting-state fMRI study. *BMC Psychiatry.* (2018) 18:339. 10.1186/s12888-018-1913-6 30340472 PMC6194586

[10] Xue SW Wang D Tan Z Wang Y Lian Z Sun Y Disrupted brain entropy and functional connectivity patterns of thalamic subregions in major depressive disorder. *Neuropsychiatr Dis Treat.* (2019) 15:2629–38. 10.2147/NDT.S220743 31571880 PMC6750201

[11] Tu PC Bai YM Li CT Chen MH Lin WC Chang WC Identification of common thalamocortical dysconnectivity in four major psychiatric disorders. *Schizophr Bull.* (2018) 45:1143–51. 10.1093/schbul/sby166 30500946 PMC6737486

[12] Kong QM Qiao H Liu CZ Zhang P Li K Wang L Aberrant intrinsic functional connectivity in thalamo-cortical networks in major depressive disorder. *CNS Neurosci Ther.* (2018) 24:1063–72. 10.1111/cns.12831 29493113 PMC6490069

[13] Preti MG Bolton TA Van De Ville D. The dynamic functional connectome: state-of-the-art and perspectives. *Neuroimage.* (2017) 160:41–54. 10.1016/j.neuroimage.2016.12.061 28034766

[14] Wu Y Zheng Y Li J Liu Y Liang X Chen Y Subregion-specific, modality-dependent and timescale-sensitive hippocampal connectivity alterations in patients with first-episode, drug-naive major depression disorder. *J Affect Disord.* (2022) 305:159–72. 10.1016/j.jad.2022.02.052 35218862

[15] Behrens TE Johansen-Berg H Woolrich MW Smith SM Wheeler-Kingshott CA Boulby PA Non-invasive mapping of connections between human thalamus and cortex using diffusion imaging. *Nat Neurosci.* (2003) 6:750–7. 10.1038/nn1075 12808459

[16] Fan L Li H Zhuo J Zhang Y Wang J Chen L The human brainnetome atlas: a new brain atlas based on connectional architecture. *Cereb Cortex.* (2016) 26:3508–26. 10.1093/cercor/bhw157 27230218 PMC4961028

[17] American Psychiatric Association. *Diagnostic and statistical manual of mental disorders.* (Vol. 21). Virginia, VA: American Psychiatric Association (2013). p. 591–643.

[18] Hamilton M. Development of a rating scale for primary depressive illness. *Br J Soc Clin Psychol.* (1967) 6:278–96. 10.1111/j.2044-8260.1967.tb00530.x 6080235

[19] Schoonheim MM Hulst HE Brandt RB Strik M Wink AM Uitdehaag BM Thalamus structure and function determine severity of cognitive impairment in multiple sclerosis. *Neurology.* (2015) 84:776–83. 10.1212/WNL.0000000000001285 25616483

[20] Wang J Wang X Xia M Liao X Evans A He Y. GRETNA: a graph theoretical network analysis toolbox for imaging connectomics. *Front Hum Neurosci.* (2015) 9:386. 10.3389/fnhum.2015.00386 26175682 PMC4485071

[21] Friston KJ Williams S Howard R Frackowiak RS Turner R. Movement-related effects in fMRI time-series. *Magn Reson Med.* (1996) 35:346–55. 10.1002/mrm.1910350312 8699946

[22] Hallquist MN Hwang K Luna B. The nuisance of nuisance regression: spectral misspecification in a common approach to resting-state fMRI preprocessing reintroduces noise and obscures functional connectivity. *Neuroimage.* (2013) 82:208–25. 10.1016/j.neuroimage.2013.05.116 23747457 PMC3759585

[23] Leonardi N Van De Ville D. On spurious and real fluctuations of dynamic functional connectivity during rest. *Neuroimage.* (2015) 104:430–6. 10.1016/j.neuroimage.2014.09.007 25234118

[24] Barber AD Hegarty CE Lindquist M Karlsgodt KH. Heritability of functional connectivity in resting state: assessment of the dynamic mean, dynamic variance, and static connectivity across networks. *Cereb Cortex.* (2021) 31:2834–44. 10.1093/cercor/bhaa391 33429433 PMC8325018

[25] Chen H Nomi JS Uddin LQ Duan X Chen H. Intrinsic functional connectivity variance and state-specific under-connectivity in autism. *Hum Brain Mapp.* (2017) 38:5740–55. 10.1002/hbm.23764 28792117 PMC5783325

[26] He C Chen Y Jian T Chen H Guo X Wang J Dynamic functional connectivity analysis reveals decreased variability of the default-mode network in developing autistic brain. *Autism Res.* (2018) 11:1479–93. 10.1002/aur.2020 30270547

[27] Li Y Zhu Y Nguchu BA Wang Y Wang H Qiu B Dynamic functional connectivity reveals abnormal variability and hyper-connected pattern in autism spectrum disorder. *Autism Res.* (2020) 13:230–43. 10.1002/aur.2212 31614075

[28] Jia H Wu X Wang E. Aberrant dynamic functional connectivity features within default mode network in patients with autism spectrum disorder: evidence from dynamical conditional correlation. *Cogn Neurodyn.* (2022) 16:391–9. 10.1007/s11571-021-09723-9 35401865 PMC8934807

[29] Yu M Linn KA Cook PA Phillips ML Mcinnis M Fava M Statistical harmonization corrects site effects in functional connectivity measurements from multi-site fMRI data. *Hum Brain Mapp.* (2018) 39:4213–27. 10.1002/hbm.24241 29962049 PMC6179920

[30] Smith SM Nichols TE. Threshold-free cluster enhancement: addressing problems of smoothing, threshold dependence and localisation in cluster inference. *Neuroimage.* (2009) 44:83–98. 10.1016/j.neuroimage.2008.03.061 18501637

[31] Wang C Wu H Chen F Xu J Li H Li H Disrupted functional connectivity patterns of the insula subregions in drug-free major depressive disorder. *J Affect Disord.* (2018) 234:297–304. 10.1016/j.jad.2017.12.033 29587165

[32] Cohen JR. The behavioral and cognitive relevance of time-varying, dynamic changes in functional connectivity. *Neuroimage.* (2018) 180:515–25. 10.1016/j.neuroimage.2017.09.036 28942061 PMC6056319

[33] Barttfeld P Uhrig L Sitt JD Sigman M Jarraya B Dehaene S. Signature of consciousness in the dynamics of resting-state brain activity. *Proc Natl Acad Sci U.S.A.* (2015) 112:887–92. 10.1073/pnas.1418031112 25561541 PMC4311826

[34] Hutchison RM Hutchison M Manning KY Menon RS Everling S. Isoflurane induces dose-dependent alterations in the cortical connectivity profiles and dynamic properties of the brain’s functional architecture. *Hum Brain Mapp.* (2014) 35:5754–75. 10.1002/hbm.22583 25044934 PMC6869297

[35] Hudetz AG Liu X Pillay S. Dynamic repertoire of intrinsic brain states is reduced in propofol-induced unconsciousness. *Brain Connect.* (2015) 5:10–22. 10.1089/brain.2014.0230 24702200 PMC4313411

[36] Shine JM Koyejo O Poldrack RA. Temporal metastates are associated with differential patterns of time-resolved connectivity, network topology, and attention. *Proc Natl Acad Sci U.S.A.* (2016) 113:9888–91. 10.1073/pnas.1604898113 27528672 PMC5024627

[37] Uddin LQ. Cognitive and behavioural flexibility: neural mechanisms and clinical considerations. *Nat Rev Neurosci.* (2021) 22:167–79. 10.1038/s41583-021-00428-w 33536614 PMC7856857

[38] McIntyre RS Xiao HX Syeda K Vinberg M Carvalho AF Mansur RB The prevalence, measurement, and treatment of the cognitive dimension/domain in major depressive disorder. *CNS Drugs.* (2015) 29:577–89. 10.1007/s40263-015-0263-x 26290264

[39] Pedersen M Zalesky A Omidvarnia A Jackson GD. Multilayer network switching rate predicts brain performance. *Proc Natl Acad Sci U.S.A.* (2018) 115:13376–81. 10.1073/pnas.1814785115 30545918 PMC6310789

[40] Ramirez-Mahaluf JP Medel V Tepper Á Alliende LM Sato JR Ossandon T Transitions between human functional brain networks reveal complex, cost-efficient and behaviorally-relevant temporal paths. *Neuroimage.* (2020) 219:117027. 10.1016/j.neuroimage.2020.117027 32522663

[41] Patel VP Feinstein A. The link between depression and performance on the symbol digit modalities test: mechanisms and clinical significance. *Mult Scler.* (2019) 25:118–21. 10.1177/1352458518770086 29648500

[42] Chu M Xu T Wang Y Wang P Gu Q Liu Q The impact of childhood trauma on thalamic functional connectivity in patients with obsessive-compulsive disorder. *Psychol Med.* (2022) 52:2471–80. 10.1017/S0033291720004328 33213536

[43] Hong A Zhou S Yang C Liu X Su S Wang Z. Impact of childhood trauma on the abnormal functional connectivity of brain regions in the fear network model of panic disorder. *J Affect Disord.* (2023) 329:500–10. 10.1016/j.jad.2023.02.128 36858271

[44] Xie H Huffman N Shih CH Cotton AS Buehler M Brickman KR Adverse childhood experiences associate with early post-trauma thalamus and thalamic nuclei volumes and PTSD development in adulthood. *Psychiatry Res Neuroimaging.* (2022) 319:111421. 10.1016/j.pscychresns.2021.111421 34864509 PMC8724406

[45] Madden RA Atkinson K Shen X Green C Hillary RF Hawkins E Structural brain correlates of childhood trauma with replication across two large, independent community-based samples. *Eur Psychiatry.* (2023) 66:e19. 10.1192/j.eurpsy.2022.2347 36697368 PMC9970154

[46] Rakesh D Kelly C Vijayakumar N Zalesky A Allen NB Whittle S. Unraveling the consequences of childhood maltreatment: deviations from typical functional neurodevelopment mediate the relationship between maltreatment history and depressive symptoms. *Biol Psychiatry Cogn Neurosci Neuroimaging.* (2021) 6:329–42. 10.1016/j.bpsc.2020.09.016 33454282

[47] Philip NS Tyrka AR Albright SE Sweet LH Almeida J Price LH Early life stress predicts thalamic hyperconnectivity: a transdiagnostic study of global connectivity. *J Psychiatr Res.* (2016) 79:93–100. 10.1016/j.jpsychires.2016.05.003 27214526 PMC4894492

[48] Kim K Kim SW Myung W Han CE Fava M Mischoulon D Reduced orbitofrontal-thalamic functional connectivity related to suicidal ideation in patients with major depressive disorder. *Sci Rep.* (2017) 7:15772. 10.1038/s41598-017-15926-0 29150619 PMC5693996

